# Corticosteroid delivery using oral mucosa equivalents for the treatment of inflammatory mucosal diseases

**DOI:** 10.1111/eos.12761

**Published:** 2021-03-01

**Authors:** Zulfahmi Said, Craig Murdoch, Jens Hansen, Lars Siim Madsen, Helen E. Colley

**Affiliations:** ^1^ School of Clinical Dentistry University of Sheffield Sheffield UK; ^2^ Faculty of Dentistry Universiti Sains Islam Malaysia Kuala Lumpur Malaysia; ^3^ AFYX Therapeutics Copenhagen Denmark

**Keywords:** drug delivery, inflammation, mouth mucosa, oral lichen planus, T‐lymphocytes

## Abstract

Oral lichen planus (OLP) is an immune‐mediated disease of the oral mucosa with idiopathic aetiology. It is frequently treated with topical corticosteroids (applied as gels, mouthwashes, or sprays); however, the mucosal exposure times of topical corticosteroids are short because of removal by the constant flow of saliva and mechanical forces. In this study we used cell monolayers, as well as oral mucosal equivalents (OMEs) containing activated T‐cells, to examine corticosteroid potency and delivery of clobetasol‐17‐propionate from a novel electrospun mucoadhesive patch. The OMEs displayed tight junctions, desmosomes, hemidesmosomes, and an efficient permeability barrier. Following application of corticosteroids to cells cultured as monolayers, the degree of cytotoxicity measured correlated to the level of potency recognized for each corticosteroid; by contrast, OMEs were largely unaffected by corticosteroid treatment. Permeation of clobetasol‐17‐propionate into and through the OMEs was time‐ and dose‐dependent, regardless of whether this corticosteroid was delivered in liquid form or from a mucoadhesive patch, and both liquid‐ and patch‐delivered clobetasol‐17‐propionate significantly reduced the secretion of interleukin‐2 by activated T‐cells. This study confirms that OMEs are more suitable models than cell monolayers for evaluating toxicity and drug delivery. After topical exposure, clobetasol‐17‐propionate accumulated in OMEs at a higher level than betamethasone‐17‐valerate and hydrocortisone‐17‐valerate, and exerted its immunosuppressive actions following application via the patch delivery system, highlighting the efficacy of this mode of drug delivery to treat OLP.

Oral lichen planus (OLP) and other closely related lichenoid lesions specifically affect the stratified squamous epithelium of the oral mucosa (1) and are relatively common, affecting 1%–2% of the world's population, with prevalence increasing with age (2). Clinically, OLP most commonly presents on the buccal mucosa, gingivae, and lateral tongue as white papules or plaques that form reticular striations, with erythema, erosion, and ulceration frequently associated (1, 3). Histological diagnosis is based on the presence of a dense mononuclear leukocyte infiltrate in the superficial lamina propria, predominantly containing CD4‐helper and CD8^+^ T‐lymphocytes, along with hyperkeratosis, atrophy of the epithelium, and degeneration of the basement membrane (4, 5).

The exact aetiology of OLP is idiopathic, and pathogenesis is mediated by dysregulated cell‐mediated immunity. Immunopathogenesis is initiated by activation of antigen‐presenting cells, most likely through antigen uptake by Langerhans cells, although macrophages have also been implicated. Increased local secretion of pro‐inflammatory mediators triggers release of the lymphocyte‐recruiting C‐X‐C motif chemokines (CXCL) 9, 10, and 11, and the C‐C motif chemokine 5 (CCL5), all of which attract T‐cell subsets into the developing lesion. Antigen‐presenting cells and oral keratinocytes present antigen to CD4+ and CD8+ T‐cells whereupon activated helper CD4+ T‐lymphocytes secrete IL‐2, IL‐12 and IFN‐γ, promoting receptor‐mediated activation of cytotoxic CD8+ T‐cells that in turn mediate apoptosis of basal keratinocytes causing pathology. Increasing evidence suggests that other T‐lymphocyte subsets, such as regulatory T‐cells, T‐helper 17 cells, and mucosal‐associated invariant T‐cells, are also implicated in OLP pathogenesis, while mast cell degranulation releases matrix metalloproteases and chymase, leading to basement membrane degradation that aids further recruitment of lymphocytes (6).

Symptomatic treatment options are mainly dependent on topical application of immune‐modulating corticosteroids, including triamcinolone acetonide and betamethasone‐17‐valerate, or halogenated forms of corticosteroid (such as clobetasol‐17‐propionate), whereas systemically delivered prednisolone is often the choice for severe lesions that do not respond to topical therapy (1, 7). Topical corticosteroids have been formulated into ointments, sprays, mouthwashes, and adhesive paste dosage forms (8, 9, 10, 11), although these are generally considered to be suboptimal as a result of rapid removal of such formulations from the lesion site by saliva flow and mechanical forces, culminating in short exposure times and unpredictable drug distribution. To circumvent this, attention has been focussed on the development of alternative mucoadhesive oral drug‐delivery systems (12, 13, 14, 15, 16). Indeed, we recently reported the fabrication of a novel dual‐layer electrospun mucoadhesive system, composed of a highly mucoadhesive inner layer and a protective, outer backing layer, specifically designed for oral mucosal drug delivery (17).

The development of new drug‐delivery devices has been hampered by the lack of *in* *vitro* model systems that accurately mimic the human oral mucosa. Oral mucosal equivalents (OMEs) based on the TR146 metastatic oral cancer cell line have previously been used but, in contrast to native oral mucosa, these malignant epithelial cells do not stratify and display altered cytokeratin profiles (18). Oral mucosal equivalents composed of normal oral keratinocytes and fibroblasts have been produced and used to evaluate drug delivery (19) but these models require access to primary cells that have a limited lifespan, so their use is restricted. We recently developed a full‐thickness OME based on immortalized oral keratinocytes and showed that this OME displayed a high degree of similarity to native tissue in terms of histological structure and marker expression (20), although detailed information about its barrier properties has yet to be provided.

This study aimed to characterize our previously established OME for barrier function and usefulness in drug delivery, and then to examine the potency of several corticosteroids in both two‐ and three‐dimensional culture systems. We subsequently compared topical delivery and activity of clobetasol‐17‐propionate from a mucoadhesive electrospun patch and when administered in liquid form, on an *in vitro* OLP‐like model consisting of OMEs with activated T‐cells.

## MATERIAL AND METHODS

All reagents were purchased from Sigma‐Aldrich and used according to the manufacturer’s instructions, unless otherwise stated.

### Cell culture

Immortalized buccal keratinocytes (FNB6‐TERT; Ximbio) (21) were cultured in flavin‐ and adenine‐enriched medium (22) and used from passages 10–20. Normal oral fibroblasts (NOFs) isolated from the buccal mucosa of a 34‐yr‐old female non‐smoker, who had given written, informed consent (ethical approval number 09/H1308/66), were cultured in Dulbecco's modified Eagle's medium (DMEM) supplemented with 10% (v/v) fetal bovine serum (FBS), 2 mM l‐glutamine, 100 IU ml^–1^ penicillin, and 100 μg ml^–1^ of streptomycin. Jurkat E6‐1 lymphocytes (American Type Culture Collection) were cultured in flavin‐ and adenine‐enriched medium without hydrocortisone. Jurkat cells were activated by treatment with phytohaemagglutinin (PHA) (5 µg ml^–1^) and phorbol 12‐myristate‐13‐acetate (PMA) (100 ng ml^–1^) for 24 h prior to use.

### Oral mucosal equivalents

The OMEs were constructed as previously described (20). Briefly, freeze‐dried rat‐tail collagen (5 mg ml^–1^), dissolved in 0.1 M acetic acid, was reconstituted in 10% (v/v) FBS, 10 × DMEM, 2 mM l‐glutamine, and reconstitution buffer (2.2% sodium bicarbonate, 4.8% HEPES, 0.248% NaOH in deionized H_2_0) and the pH was adjusted to 7.4 to give a final collagen concentration of 3.5 mg ml^–1^. Then, 2.5 × 10^5^ NOFs ml^–1^ were added to the collagen solution and 1 ml was transferred to 12‐mm‐diameter, 0.4 μm pore size, cell culture transwell inserts (Millipore) and incubated at 37°C for 2 h to allow the collagen to gel before being submerged in flavin‐ and adenine‐enriched medium and incubated for 2 d at 37°C. FNB6‐TERT keratinocytes (2.5 × 10^5^ cells per model) were seeded onto the surface of the NOF‐populated collagen gel and incubated, submerged, for a further 5 d at 37°C. Oral mucosal equivalents were then raised to an air‐to‐liquid interface at 37°C, in an atmosphere of 5% CO_2_, and cultured for 10 d at the same temperature and atmosphere, with a change in medium every 2 d. To generate a T‐cell inflammatory OME, 2 × 10^6^Jurkat cells activated (as described in the previous paragraph) for 24 h with PHA and PMA were added to the basolateral compartment of the transwell before use in experiments.

### Histological and immunohistochemical analyses

For histological analysis, OMEs were fixed in 10% nuetral‐buffered formalin overnight and subjected to routine histological processing. Formalin‐fixed, paraffin‐embedded oral mucosal tissue was used as a control; ethical approval was granted by the Sheffield Research Ethics Committee (Ref: 07/H1309/105). For immunohistochemistry, tissue sections (5 µm in thickness) were dewaxed, rehydrated through a series of alcohol dilutions, then incubated for 20 min in 3% hydrogen peroxide in methanol to neutralize endogenous peroxidases. Antigen retrieval was achieved using 0.01 M citrate buffer (pH 6) at high temperature. Following blocking with normal goat serum for 20 min, sections were incubated with anti‐claudin 4 (EPRR17575; 1:4000), anti‐E‐cadherin (HECD‐1; 1:200), or IgG isotype control primary antibodies for 1 h at 20°C. Secondary antibody and avidin–biotin complex (ABC) (Vectastain Elite ABC kit; Vector Laboratories) were used in accordance with the manufacturer's instructions. Finally, 3’‐diaminobenzidine tetrahydrochloride (DAB; Vector Laboratories) was used to visualize peroxidase activity and the sections were counterstained with Harris's haematoxylin.

### Transmission electron microscopy analysis

The OMEs were cut into 2 mm thick sections and fixed in 3% glutaraldehyde diluted in 0.1 M cacodylate buffer, at pH 7.4, for 2 h at 4°C before rinsing twice in 0.1 M phosphate buffer (pH 7.4) at 4°C. Then, the OME sections were post‐fixed in 2% osmium tetroxide for 2 h at room temperature, rinsed in 0.1 M phosphate buffer, dehydrated in an ethanol gradient with propylene oxide for 15 min at 20°C, and incubated in propylene oxide/araldite resin (1:1; v/v) overnight. The following day, excess resin mixture was removed by evaporation for 1 h, after which the OME sections were incubated in 100% araldite resin for 6 h and then embedded in fresh araldite resin for 72 h, all at 20°C. Sections (70–90 nm in thickness) were stained with 3% uranyl acetate and Reynold's lead citrate. The sections were examined using an FEI Tecnai TEM at an accelerating voltage of 80 kV and images were taken using a Gatan digital camera.

### OME viability

Analysis of cell metabolism using alamarBlue (ThermoFisher Scientific) was used as a surrogate measurement for OME viability. alamarBlue solution (10% [v/v] in flavin‐ and adenine‐enriched medium) was added to each insert and incubated for 5 h, after which the fluorescence intensity of the medium was measured using an excitation wavelength of 550 nm and an emission wavelength of 590 nm.

### Transepithelial electrical resistance analysis

Oral mucosal equivalents were pre‐incubated for 15 min in PBS to stabilize the electrical condition of the cells. Then, transepithelial electrical resistance (TEER) values were measured using an Epithelial Volt Ohm Meter (World Precision Instruments) following Ohm's (Ω) law. The TEER across the OME was obtained by subtracting the TEER value derived from a blank transwell. An OME treated with 5% SDS was used as a positive control. Epithelial resistance (Ω cm^–2^) was calculated using the equation: TEER = Resistance (Ω) × tissue surface area (cm^2^) (23).

### OME permeability

Fluorescently‐labelled dextrans (Thermofisher Scientific) with molecular mass values of 3, 10, and 70 kDa (250 µg ml^–1^ in Hanks’ balanced salt solution) were added to the epithelial surface of OMEs and incubated at 37°C in an atmosphere of 5% CO_2_. Oral mucosal equivalents treated with PBS or 5% SDS acted as negative and positive controls, respectively. After 1 h, the amount of dextran in the basolateral compartment was measured using a spectrometer with excitation at 495, 548, and 590 nm and emission at 525, 578, and 620 nm, respectively. Permeability was calculated by determining the percentage of total dextran in the basolateral compartment normalized to blank inserts.

### Cytotoxicity of corticosteroids on oral keratinocytes, fibroblasts, and OMEs

The cytotoxic effects of seven corticosteroids – hydrocortisone‐17‐butyrate (HB), hydrocortisone‐17‐valerate (HV), triamcinolone acetonide (TA), betamethasone‐17‐valerate (BV), betamethasone‐17,21‐dipropionate (BD), budesonide (BU), and clobetasol‐17‐propionate (CP) (0.01–500 µM) – on FNB6 and NOF (5 × 10^4^ cells per well) monolayers were assessed by determining the half maximal inhibitory concentration (IC_50_) at 72 h using an MTT assay, as previously described (24). The cytotoxicity of CP, BV, and HV on OMEs was evaluated according to the Organization for Economic Co‐operation and Development guidelines, as described previously (25).

### Mucoadhesive patch formulations

Mucoadhesive patches (a kind gift from AFYX Therapeutics) were manufactured as previously described (17). Briefly, patches were fabricated by electrospinning polyvinylpyrrolidone (BASF, UK), Eudragit RS100 (Evonik Industries, Germany), and polyethylene oxide with a polycaprolactone backing layer (both Sigma‐Aldrich, UK). Thereafter, CP was loaded at 1, 5, or 20 µg per patch.

### Evaluation of corticosteroid permeation through OMEs

The OME‐ and OLP‐like models were exposed to topical corticosteroids (in either liquid or patch formulations) and incubated for up to 24 h. After exposure, the receptive media in the lower well of the transwell chamber was collected and the models were washed, weighed, disaggregated in collagenase (2 mg ml^–1^), and centrifuged at 500 *g* for 5 min. The supernatants were collected and the presence of corticosteroids was determined using high performance liquid chromatography (HPLC). The concentration of corticosteroid recovered in the models was calculated by normalizing to the weight of the tissue and is expressed as nM mg^–1^ of tissue.

### High performance liquid chromatography

Media and tissue analyses were performed with a Waters 2690 HPLC instrument, using a Zorbax RX‐C18 250 mm × 4.6 mm column and a mobile phase composed of acetonitrile (ACN)/water: CP and BV (45% ACN in water for 15 min, increasing to 100% ACN after 16 min) and HV (40% ACN in water for 20 min, increasing to 100% ACN after 30 min), all at 1 ml min^–1^. Emission of UV light was detected using a Waters 486 UV detector at 240 nm (for CP and BV) and 244 nm (for HV) and the concentrations of these corticosteroids was calculated from standard calibration curves.

### Enzyme‐linked immunosorbent assay

At each study time point, 500 µl of medium from the basolateral compartment was removed from MOE‐containing activated T‐cells and replaced with 500 µl of fresh medium. The samples were centrifuged (500 *g*, 5 min) and secretion of IL‐2 was evaluated using ELISA (OptEIA; BD Bioscience), according to the manufacturer's instructions.

### Statistics

All data are expressed as mean ± SD of at least three independent experiments performed in triplicate technical repeats, unless otherwise stated. Statistical analysis was undertaken using GraphPad Prism (v9.0, GraphPad Software). Pairwise comparisons were performed using the Student’s *t*‐test, and group‐wise comparisons were performed using one‐way ANOVA with Tukey's multiple comparisons test. Differences between groups were considered significant when *P* < 0.05.

## RESULTS

### Characterization of OMEs

**FIGURE 1:**
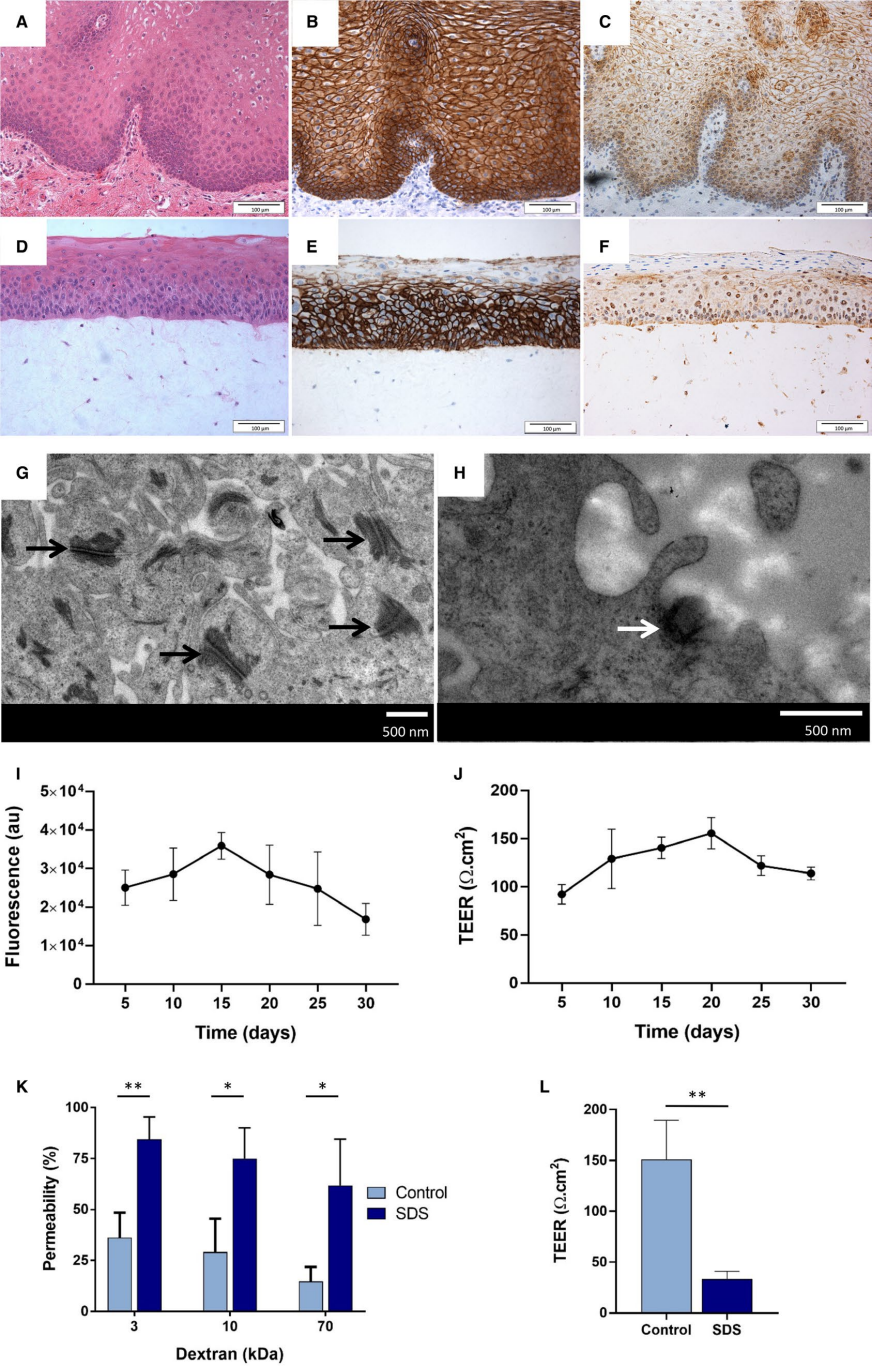
Characterization of oral mucosal equivalents (OMEs). Representative images of normal oral mucosa (A–C) and OMEs (D–F) stained using haematoxylin and eosin (H&E) (A, D) and immunohistochemically for E‐cadherin (B, E) and claudin‐4 (C, F) (scale bar = 100 µm). Transmission electron micrographs of the cellular ultrastructure of an OME show desmosomes (black arrows) (G) and a hemidesmosome (white arrow) (H) (scale bar = 500 nm). The viability and integrity of OMEs was assessed, over 30 d in culture, using a metabolic activity assay (alamarBlue) (I) and transepithelial electrical resistance (TEER) (J). Permeability was measured by evaluating permeation of 3‐, 10‐, and 70‐kDa dextrans applied topically to the models for 1 h (control) compared to treatment with 5% SDS (K) and by using TEER (L). Data are expressed as mean ±SD of three independent experiments. Statistics were performed using the Student's *t*‐test; *P < 0.05 and **P < 0.01.

Full‐thickness, tissue‐engineered OMEs were generated using immortalized buccal keratinocytes (FNB6‐TERT). Histological analysis revealed that on day 10, the OMEs produced a multilayered, stratified epithelium, approximately 210 µm in thickness. Immunohistochemistry analyses demonstrated that cell membranes throughout the epithelium stained positive for E‐cadherin and claudin‐4, and that the nucleus of keratinocytes also stained positive for claudin‐4, reflecting the findings observed *in vivo* (Figure 1A‐F). Further examination of the cellular ultrastructure using transmission electron microscopy identified desmosomes and hemidesmosomes (Figure 1G,H), confirming the presence of cell–cell and cell–basement membrane attachments. Metabolic activity of the models increased over the first 15 d in culture before slowly decreasing until day 30 (Figure 1I). This was consistent with TEER, which increased from 92 ± 7.1 Ω cm^–2^on day 5 to 156 ± 11 Ω cm^–2^ on day 20, before decreasing to 114 ± 4.7Ω cm^–2^ on day 30 (Figure 1J). Barrier properties were further tested using fluorescent dextran. After 1 h of incubation, OMEs demonstrated permeability of only 36 ± 12%, 29 ± 16%, and 15 ± 7% for the 3‐, 10‐, and 70‐kDa dextrans, respectively. However, for OMEs treated with 5% SDS a significant increase of permeability, to 84 ± 11%, 74 ± 15%, and 61 ± 23% for the 3‐, 10‐, and 70‐kDa dextrans, respectively, was observed (Figure 1K), and a reduction in TEER from 151 ± 39 to 33 ± 8 Ω cm^–2^ (Figure 1L).

### *In vitro* cytotoxicity of commonly used corticosteroids

To determine the *in vitro* cytotoxicity of corticosteroids, oral keratinocytes (FNB6) were exposed to seven commonly used corticosteroids, and the IC_50_ of each corticosteroid was measured 72 h after exposure. The cytotoxicity (in order from high to low) was: CP>BU>BD>BV>TA>HV>HB. A similar pattern was seen for NOFs, of: CP>BD>BV>BU>HB>TA>HV (Table 1). The toxicities of CP, BV, and HV were further tested using OMEs, and their IC_50_ values were all found to be greater than 400 nM. Corticosteroids with high, medium and low cytotoxicity – CP, BV, and HV, respectively – were selected for use in further experiments.

**TABLE 1:** Cytotoxicity of different corticosteroids on keratinocyte (FNB6) and normal oral fibroblast (NOF) monolayers.

Corticosteroid	FNB6 (nM) (mean ± SD)	NOF (nM) (mean ± SD)
Hydrocortisone‐17‐butyrate	340.9 ± 20.3	246.4 ± 29.4
Hydrocortisone‐17‐valerate	261.3 ± 28.9	314.3 ± 52.1
Triamcinolone acetonide	194.8 ± 41.6	259.5 ± 24.3
Betamethasone‐17‐valerate	92.9 ± 17.3	103.7 ± 7.2
Betamethasone‐17,21‐dipropionate	59.2 ± 5.9	74.8 ± 1.71
Budesonide	46.4 ± 5.2	150.9 ± 17.7
Clobetasol‐17‐propionate	32.3 ± 0.9	40.3 ± 2.1

Susceptibility of FMB6 and NOF monolayers to the cytotoxic effects of corticosteroids was evaluated by measuring the half maximal inhibitory concentration (IC_50_) for each corticosteroid, using an MTT assay, 72 h after exposure.

### Corticosteroid permeation into OMEs

**FIGURE 2:**
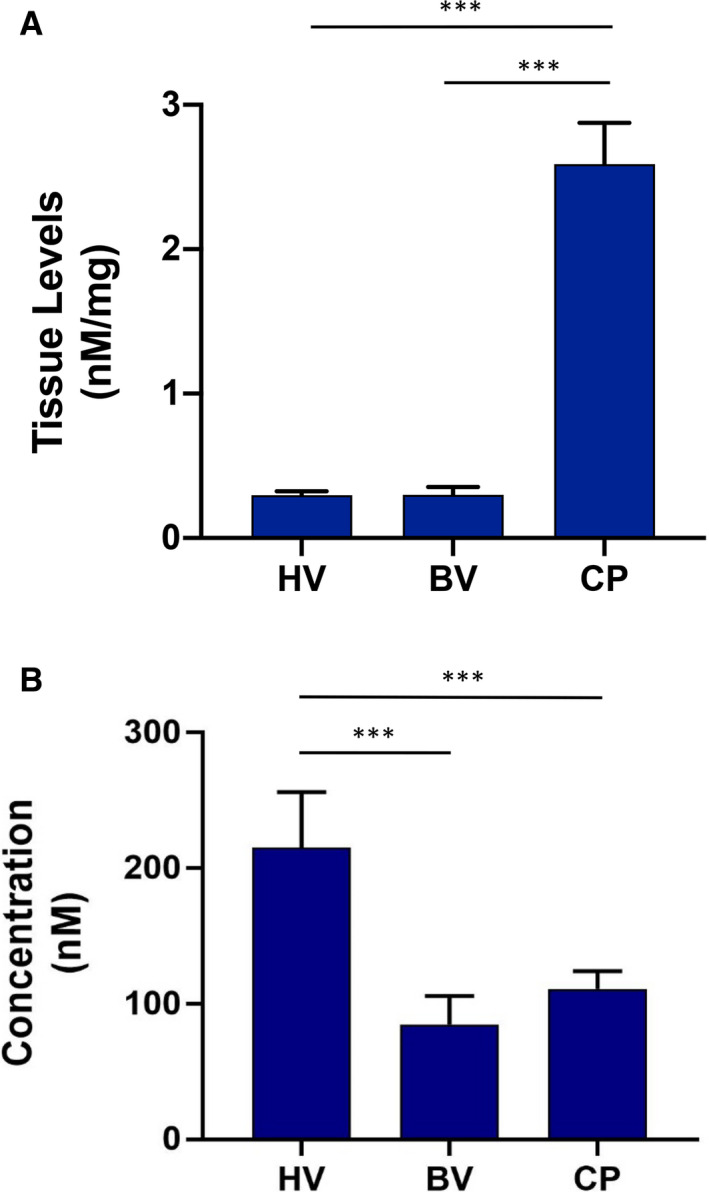
Permeation of oral mucosal equivalents (OMEs) by corticosteroids. Permeation of tissue (A) and receptive media (B) was measured after 1 h of exposure to clobetasol‐17‐propionate (CP), betamethasone‐17‐valerate (BV), and hydrocortisone‐17‐valerate (HV) (all 5 µM). Data are expressed as mean ±SD of three independent experiments. Statistics were performed using one‐way ANOVA with Tukey's post‐hoc multiple comparisons test; ***P < 0.001.

Next, CP, BV, or HV (5 μM) were delivered topically to the OMEs and the concentration that accumulated in the tissue and receptive medium after 1 h was determined by HPLC. We found that CP accumulated in tissue at a significantly higher level (2.6 ± 0.3 nM mg^–1^) than either BV or HV (0.3 nM mg^–1^) (Figure 2A). By contrast, HV was found to permeate through the models and accumulate in receptive medium at a significantly higher concentration (215 ± 41 nM) than either BV (85 ± 21 nM) or CP (111 ± 13 nM) (Figure 2B). As a result of high levels of accumulation in tissue, CP was used in further experiments.

### Delivery of CP from mucoadhesive patch into OME

**FIGURE 3:**
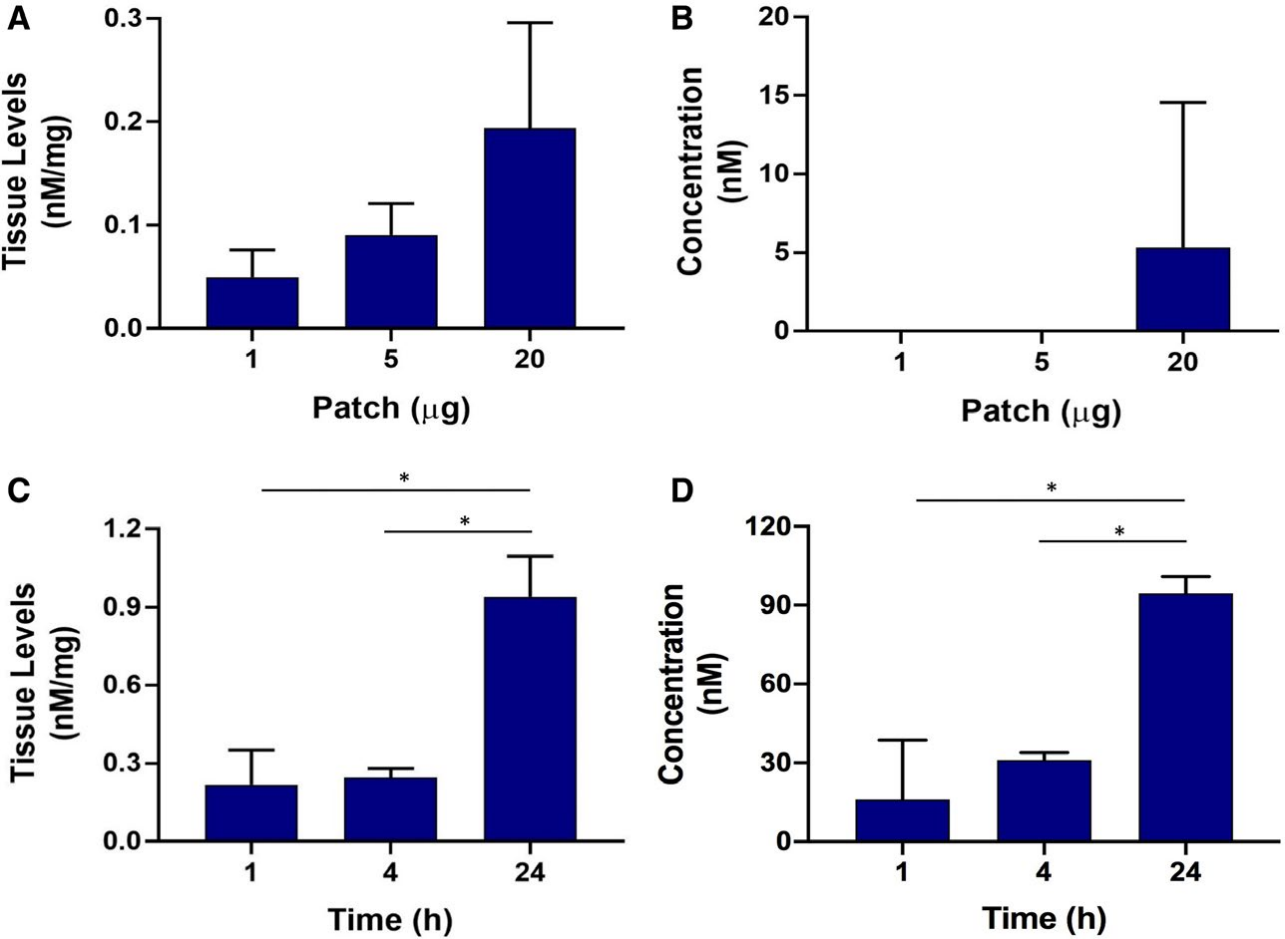
Permeation of oral mucosal equivalents (OMEs) and the receptive medium by patch‐formulated clobetasol‐17‐propionate (CP), according to concentration of CP (A, B) and duration of exposure to the patch (C, D). Permeation of OMEs (A) and the corresponding receptive media (B) by 1, 5, and 20 µg of CP (from a loaded patch) was stopped after 1 h, and the levels of CP in tissue and the concentration of CP in receptive media were measured. Permeation of tissue (C) and receptive media (D) by CP (from a patch containing 20 µg of CP) was assessed after 1, 4, and 24 h. Data are expressed as mean ±SD of three independent experiments. Statistics were performed using one‐way ANOVA with Tukey's post‐hoc multiple comparisons test; *P < 0.05.

To control corticosteroid delivery into the oral mucosa, CP was loaded into mucoadhesive patches at 1, 5, or 20 µg, applied topically to the OME for 1 h, then the levels of CP in tissue and receptive media were determined by HPLC. A dose–response effect was observed, with 0.05 ± 0.03, 0.09 ± 0.03, and 0.19 ± 0.1 nM mg^–1^ of CP accumulating in the tissue after delivery from patches containing 1, 5, or 20 µg of CP, respectively (Figure 3A). Only CP released from the patch containing 20 µg of this corticosteroid was found to accumulate in receptive medium, and the concentration was variable, with 5.3 ± 9.2 nM CP detected after 1 h (Figure 3B). As the patch containing 20 µg of CP showed the best permeation of this corticosteroid, a time–response course was performed to investigate if more CP penetrated with increased duration of exposure. No increase in CP concentration was found in tissue or receptive medium when the patch was applied topically for 4 h but a significant increase of CP was observed when the exposure time was increased to 24 h, with 0.94 ± 0.16 nM mg^–1^ of CP found in tissue and 95 ± 6.4 nM CP found in receptive medium, demonstrating that a longer contact time of the patch allows a larger quantity of CP to be released (Figure 3C,D).

### Delivery of CP from mucoadhesive patch into an OME OLP‐like model

**FIGURE 4:**
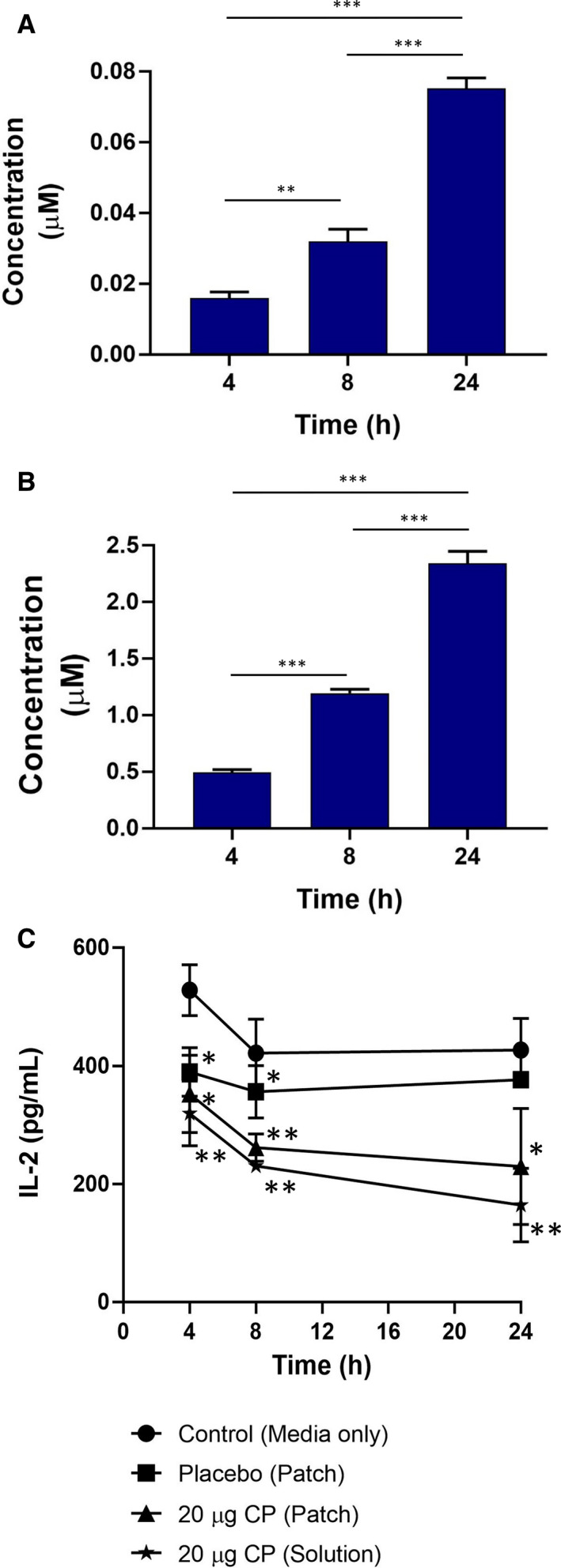
Permeation of receptive medium by clobetasol‐17‐propionate (CP) from a patch loaded with 20 µg of CP (A) and from a solution also containing 20 µg of CP (B) was assessed after 1, 4, and 24 h. (C) Oral mucosal equivalents (OMEs) containing activated T‐cells were treated with medium alone (control), placebo (patch only), a patch loaded with 20 µg of CP (loaded‐patch), or a solution containing 20 µg of CP (solution). After 4, 8, and 24 h, permeation of CP was evaluated as the amount of interleukin‐2 (IL‐2) released from activated T‐cells. Data are expressed as mean ±SD of three independent experiments. Statistics were performed using one‐way ANOVA with Tukey's post‐hoc multiple comparisons test compared with all groups (A, B) or the medium‐only control (C); *P < 0.05, **P < 0.01, ***P < 0.001.

To evaluate activity of CP once delivered through the OME, a T‐cell inflammatory OME was developed. Jurkat cells treated with PHA and PMA secreted IL‐2 in a time‐dependent manner, with maximal secretion (1626.3 ± 311.4 pg ml^–1^) achieved after 24 h. Therefore, following 2 h of stimulation, activated Jurkat cells were added to the basolateral compartment, then CP (20 µg) in patch or liquid form was added topically to the OLP‐like model and the concentrations of CP reaching the Jurkat cells in the receptive medium were determined after 4, 8, and 24 h. We found that CP, delivered from a patch, accumulated in a time‐dependent manner, from 0.016 ± 0.0017 µM at 4 h, to 0.032 ± 0.0035 µM (*P* < 0.01) at 8 h, and to 0.075 ± 0.0029 µM (*P* < 0.001) at 24 h. Delivery of CP in liquid form resulted in significantly higher concentrations of CP in receptive medium than when CP was delivered via the patch; similarly to patch delivery, the concentration of CP also increased in a time‐dependent manner when delivered in liquid form. After 4 h, 0.49 ± 0.025 µM CP was found to have penetrated the model to the basolateral compartment, with a significant increase to 1.2 ± 0.036 and 2.3 ± 0.1 µM CP after 8 and 24 h, respectively (Figure 4A,B). Release of IL‐2 from activated Jurkat cells was significantly decreased in a time‐dependent manner in response to treatment with CP, delivered either as liquid or from a patch, compared to treatment with a placebo patch or with medium alone (Figure 4C).

## DISCUSSION

The use of full‐thickness OMEs in research has increased in recent years as a result of the many advantages they offer over monolayer cell culture. The use of immortalized cells in OMEs dispenses with the requirement for a continual supply of oral tissue for primary cell isolation, but the structural and biophysical properties of these models still need to be validated. We previously showed that OMEs based on FNB6‐TERT2 immortalized oral keratinocytes display differentiation and proliferation marker profiles similar to those of native human oral mucosa (20). Further characterization now shows that these OMEs adhere tightly to the connective tissue via hemidesmosomes and display abundant cell–cell contacts, such as E‐cadherin‐containing desmosomes, and, for the first time in OMEs, claudin‐4 tight junctions, which are the major determinant of paracellular permeability (26). Interestingly, claudin‐4 expression was also localized to the keratinocyte nuclei in OMEs and human oral mucosa. This expression pattern has not been reported in oral mucosa previously, although both claudins 1 and 4 have been shown to localize to the nucleus in colon and endometrial cells, respectively, where they are believed to be involved in the transcriptional control of E‐cadherin expression (27).

The presence of an effective epithelial permeability barrier was further substantiated by TEER analysis of the OME in the present study, which revealed a higher value (156 ± 11 Ω cm^–2^ on day 20) than those found for multilayered primary oral keratinocyte‐only (134.8 ± 7.0 Ω cm^–2^) (28)) or TR146 oral cancer cell (68.2 ± 2.3 Ω cm^–2^) models (29)). Furthermore, FNB6‐based OMEs were more permeable to 3‐kDa dextran than to 70‐kDa dextran, a finding similar to that for porcine buccal mucosa for which dextran permeability was shown to occur via the paracellular route (30). Taken together, our data show that FNB6‐based OMEs display an appropriate permeability barrier and therefore are ideally suited for use in drug‐delivery studies.

Corticosteroids, often used for the topical treatment of OLP, are categorized based on their potency using the vasoconstrictor or psoriasis assay, the gold standards in determining the efficacy and anti‐inflammatory actions of these drugs (31, 32). However, *in vitro* cytotoxicity assays based on reduction of tetrazolium dyes are used to measure irritation of tissue‐engineered skin by compounds (Organisation for Economic Co‐operation guideline 3 and European Centre for the Validation of Alternative Methods) and hence may be useful for assessing corticosteroid potency *in vitro*. Based on IC_50_ values, we found corticosteroid potency on oral keratinocyte monolayers to occur in the order (high to low) CP>BU>BD>BV>TA>HV>HB, which is broadly in agreement with current corticosteroid potency criteria for both British and US national formularies (33, 34). Interestingly, IC_50_ values were much higher for OMEs than for monolayers, with the difference probably being the stratified nature of OMEs and differences in expression of xenobiotic metabolizing enzymes that are potentially increased in three‐dimensional compared with two‐dimensional cell culture, as observed for skin (35, 36).

When applied to OMEs, significantly more CP than BV or HV was found to accumulate in the mucosa, whereas HV permeated most quickly though the OME into the receptive medium of the transwell culture system. The physicochemical properties of corticosteroids dramatically influence their absorption by tissue. In general, molecules with a molecular mass of <0.5 kDa, such as corticosteroids, are easily transported through stratified epithelium (37). Esterification and addition of functional groups, such as propionate, enhances corticosteroid lipophilicity, increasing their absorption by tissue and enabling retention within the mucosa (38), which may explain why CP exhibited higher accumulation in tissue than BV and HV.

With high potency and increased retention in tissue, CP is frequently the choice for the therapeutic management of OLP (1), and so we selected this corticosteroid for incorporation into a novel electrospun mucoadhesive oral patch intended for localized, unidirectional delivery to the oral mucosa (25). Upon release from oral patches after 1 h, accumulation of CP in OMEs was dose‐dependent, whereas accumulation of CP in the receptive medium was only observed for the oral patches loaded with 20 µg of CP. In addition, following application to OMEs of oral patches loaded with 20 µg of CP, accumulation of CP increased over 24 h, both within the tissue and in the receptive medium, indicating that therapeutic levels of CP are most likely to be achieved by a patch containing 20 µg of CP. A similar trend, of accumulation of CP in tissue following use of these patches, was observed in porcine oral mucosa (25). Studies using other nanoparticle drug‐delivery systems similarly showed increased accumulation of CP in porcine skin epidermis, particularly in the stratum corneum, over a 6‐h period, although in these studies no CP was detected in the receptive medium of the Franz chamber (39, 40). These studies indicate that CP may preferentially accumulate in the most apical, keratinized surfaces of skin, whereas in non‐keratinized epithelium (such as the buccal mucosa), CP is free to penetrate into and across the epithelium. More CP was observed in the OMEs and receptive medium over 24 h when applied in liquid form than in patch form. In the soluble form, CP is freely mobile, which allows fast and direct paracellular absorption, whereas in the electrospun patch, CP is held within the polymer fibres in an amorphous form, and hydration and collapse of the polymer complex is required for CP to be released (25). Therefore, patch‐loaded drugs tend to be delivered at lower levels, and more slowly, than drugs in free solution (12, 41). Indeed, the ability to control the rate of drug release over time is a distinct advantage for treating oral lesions.

T‐cells are the main protagonists in mediating pathogenesis in OLP. Interleukin‐2 is amongst a number of important cytokines that are implicated in the disease process and it drives T‐cell activation in an autocrine manner via the IL‐2 receptor. High levels of IL‐2 have been reported in OLP lesions (42, 43), whilst corticosteroids decrease expression of the *IL2* gene and secretion of the IL‐2 protein by interfering with the transcription factors nuclear factor of activated T‐cells, activator protein 1, and nuclear factor kappa‐light‐chain‐enhancer of activated B cells (44, 45, 46, 47), hence the use of corticosteroids in treatment of OLP. Here, we used Jurkat T‐cells as a rudimentary *in vitro* model cell system to assess if the immunosuppressive effects of CP are retained when it is released from the mucoadhesive patch. Incubation of Jurkat cells with a combination of PHA and PMA directly activates the protein kinase C signalling pathway, increasing transcription of *IL2* and secretion of IL‐2 without the need for T‐cell‐receptor engagement (48). The efficiency of CP in reducing IL‐2 levels secreted by PHA/PMA‐activated T‐cells residing in the basolateral transwell chamber of the *in vitro* model of OLP was comparable between patch‐loaded CP and that delivered in solution, despite more CP entering the basolateral lower chamber when administered by solution than by patch. Therefore, it appears that the smaller amount of CP delivered from the patch was still sufficient to inhibit transcription of *IL2* in stimulated T‐cells, reflecting the high potency of CP.

A potential limitation of this study is the use of the Jurkat T‐cell lymphoblast cell line and its artificial activation using chemical stimulants. Although useful in this system as a lymphocyte source of IL‐2 production, these cells do not fully replicate the T‐helper 1 cell phenotype that dominates in OLP, let alone the other T‐cell subsets present in these lesions. Further in‐depth analysis of patch‐delivered CP‐mediated therapy would require more advanced tissue‐engineered OLP models containing appropriate T‐cell‐receptor‐complex‐activated, T‐helper 1‐polarized primary T‐cells. Such systems are currently under development. The experiments outlined in this study were also performed over a relatively short period of time and OLP is treated over a prolonged period of time, so examination of the long‐term effects of patch‐delivered therapy is warranted in future studies.

We chose to incubate the OME T‐cell model with a mucoadhesive patch for 24 h because in this model the CP has to penetrate through the entire thickness of the OME and polycarbonate filter to reach the activated T‐cells in the receptive medium, a distance of approximately 700 μM. It could be argued that this is a considerable length of time for a patch to be in contact with the oral mucosa for drug delivery. However, it must be noted that in patients with OLP, the target lymphocytes are located between the basal or suprabasal keratinocytes and just below the basement membrane in the lamina propria in mucosal tissue, which often has a denuded epithelium. Therefore, in these patients CP incorporated within an oral patch may only need to penetrate 30–40 μM to reach its target site, which thus can be reached within much shorter time periods than in the OME model described in the present study.

It is difficult to compare the mucoadhesive system used this study directly with other steroid‐containing delivery formulations. Other experimental systems are scarce (8, 49), with most studies focussed on combining corticosteroids with Orabase (9, 10, 11, 50, 51). The majority of these studies are centred on clinical trials and contain little or no data on preclinical or *in vitro* evaluation, such as drug‐release profiles or tissue penetration. The recently published Cochrane Systematic Review suggests that adhesive gel‐based corticosteroids do offer better treatment than placebo, although the authors concede that the small numbers of patients in most studies make the confidence in this finding low (7). To date, the data generated for the mucoadhesive oral patches described in this study are preclinical, making any comparisons with the Orabase formulations problematical. However, our mucosal patch delivery system has very recently completed phase II clinical trials (clinicaltrials.gov/show/NCT03592342), which will enable a direct comparison with other current formulations.

In conclusion, to the best of our knowledge, this is the first study to show the successful transmucosal delivery of a corticosteroid using electrospun patch technology, and importantly, to confirm that the drug delivered retains its anti‐inflammatory activity against activated T‐cells. This new mucoadhesive patch technology can deliver therapy directly to diseased oral tissue and therefore holds great promise for targeted, localized delivery for the treatment of OLP or for the delivery of other agents, such as anaesthetics or analgesics, which require specific, controlled delivery directly to oral tissue. The development of mucoadhesive patches will be further enhanced by improved preclinical screening using validated tissue‐engineered OMEs based on immortalized keratinocytes that are more standardized and accessible to the scientific community.

## CONFLICT OF INTEREST

LSM is chief operating officer and JH a shareholder of AFYX Therapeutics, where AFYX have translated mucoadhesive electrospun patch technology for clinical use and have intellectual property (international patent application WO 2017/085262). HEC and CM have previously received funding from AFYX Therapeutics.

## AUTHOR CONTRIBUTION

**CM**, **HEC**, and **JH** conceived the research. **CM**, **HEC**, and **JH** planned experiments. **ZS** performed the experiments whilst **JH** and **LSM** provided essential resources. **ZS**, **HEC** and **CM** analysed the data, conducted statistical analysis, interpreted the results and wrote the manuscript draft. Further manuscript editing was performed by **JH** and **LSM**.
